# Experimental Study of a Sequential Membrane Process of Ultrafiltration and Nanofiltration for Efficient Polyphenol Extraction from Wine Lees

**DOI:** 10.3390/membranes14040082

**Published:** 2024-03-30

**Authors:** Miguel-Jorge Reig-Valor, Javier Rozas-Martínez, Alexis López-Borrell, Jaime Lora-García, María-Fernanda López-Pérez

**Affiliations:** Instituto de Seguridad Industrial, Radiofísica y Medioambiental (ISIRYM), Universitat Politècnica de València (UPV), Plaza Ferrándiz y Carbonell, s/n, 03801 Alcoy, Spain; jrozmar@epsa.upv.es (J.R.-M.); allobor1@epsa.upv.es (A.L.-B.); jlora@iqn.upv.es (J.L.-G.); malope1@iqn.upv.es (M.-F.L.-P.)

**Keywords:** ultrafiltration, nanofiltration, polyphenols, fouling, wine lees, revalorization

## Abstract

The wine industry is a sector of great importance in the Spanish economy, contributing substantial annual revenues. However, one challenge facing the industry is the amount of waste generated, reaching millions of tons annually. These residues consist of organic matter of industrial interest, such as polyphenols. These substances are characterised by their excellent antioxidant properties, making them ideal for use in the food, cosmetic, and pharmaceutical industries. Modern techniques, such as membrane technology, are explored for their extraction based on separating compounds according to size. This work studies a sequential filtration process using ultrafiltration (UF) and nanofiltration (NF) membranes at different operating conditions (2 bar and 9.5 bar for UF and NF, respectively, at 20 °C) to extract polyphenols from wine lees. The results show a total polyphenols rejection rate for each process of 54% for UF and 90% for NF. Pore blocking models have been studied for the UF process and an intermediate pore blocking of the membrane upon wine lees filtration has been identified. A mathematical model that justifies the behavior of a polymeric NF membrane with the filtration of pre-treated vinasse residues has been validated. This study shows a viable process for extracting polyphenols from wine lees with sequential membrane technology.

## 1. Introduction

### 1.1. Polyphenols: Antioxidants for the Industry

Polyphenols are chemical compounds found in various plants and vegetables in nature, characterised by a chemical structure that includes phenolic groups. These phenolic groups are the main reasons why polyphenols acquire excellent antioxidant properties, which protect cells from the attack of free radicals, enabling them to avoid their oxidation and deterioration [1].

Moreover, these compounds present other properties that make them even more appealing for specific industries, such as the pharmaceutical, cosmetic, and food industries. These attributes include excellent anti-inflammatory, cardioprotective, anticarcinogenic, antiaging and antimicrobial properties [2,3].

Currently, more than 10,000 species have been discovered and identified, which can be classified according to different criteria, including their origin, distribution in nature or chemical structure, among others [4]. Flavonoids, the most voluminous and studied group, consist of 8000 known species organized into 13 classes based on the type of heterocyclic ring present. Flavonoids are present in various foods, including fruits and vegetables such as blueberries, grapes, green tea, and olives [5,6].

There is substantial interest in these compounds as high value–added components [7,8], and their obtention is based on extraction from fruits and vegetables or chemical synthesis [9]. One potential source of polyphenols is grapes, a fruit rich in these compounds [10]. In addition, during the alcoholic fermentation of wine from grapes, compounds such as tyrosol and hydroxytyrosol are synthesized, which are two of the most valued species due to their high antioxidant properties [11].

### 1.2. Revalorization of Residues from the Wine Industry

The wine production industry in Spain is a tremendously important sector for the economy, society, and culture. In fact, in 2023, it was estimated that around 30.74 million hectoliters were produced in the country [12], making it the world’s third-highest producer of the beverage [13].

Furthermore, the industry is well known for generating large amounts of residue, known as wine lees, which can be challenging to process or obtain any value from. It is estimated that around 5 or 6 million tonnes of wastage is produced annually during the grape harvest stage [14]. Although these residues are currently used to obtain animal food, it is possible to revalorize them, making them more valuable to industries.

Therefore, given the increasing change in mentality towards sustaining environmentally friendly industries and reducing the residues generated, the wine production industry is an exciting source of high-value products for many sectors [15].

### 1.3. Membrane Technology for Polyphenol Extraction

Several techniques have been developed to extract polyphenols from fruits, such as liquid-liquid and solid-solid extraction, ultrasound-assisted extraction, microwave extraction, or supercritical fluid extraction [2,16]. However, membrane separation technology emerges as a more sustainable alternative due to its lack of solvent usage, reduced energy cost, and easy assembly based on modules [17,18].

The technologies involved in extracting polyphenols from grapes include different processes depending on the substances treated and the operating conditions [19]. Microfiltration (MF) and ultrafiltration (UF) work at low pressures (0.5 to 2 bar and 0.5 to 7 bar, respectively) and are used to separate voluminous organic particles and even bacteria. Nanofiltration (NF) is the next category of membrane processing technology; it operates at modest pressures (5 to 10 bar) and is more selective when separating compounds of a specific size. Finally, reverse-osmosis (RO) processes work at high pressures (5 to 80 bar) and are primarily used for water desalination.

Other authors have studied these technologies to extract polyphenols from different fruits and sources, such as brine from olive production, olive oil washing wastewater, and bergamot juice [20,21,22]. Regarding the winemaking industry, the use of membrane technology to extract polyphenols from biproducts has been widely studied, including the purification of grape marc phenolic compounds using a sequential UF and NF process [23]. However, the most significant residues come from pomace, grape seeds and lees [24], which represent the residues on which UF and NF processes can be implemented most effectively and which have been intensively studied by other authors [25,26,27,28]. Due to the nature of wine lees, they do not require the use of any solvents to be directly suitable for membrane filtration, thereby resulting in lower costs for industries. Following previous studies, using a sequential membrane system by introducing a pretreatment step using a UF filtration process could help reduce the fouling effect on an NF membrane for extracting the desired phenolic compounds from wine lees, making it more selective and extending its shelf life.

In membrane processes, it is common to work with a set of parameters that adequately define any system. The permeate flux density ($J_{v}$) is described according to Darcy’s law in Equation (1).

(1) $$J_{v}=L_{p}\cdot ∆P=\frac{∆P}{\mu \cdot R_{m}}=\frac{V_{p}}{A_{m}\cdot t}$$

where $L_{p}$ is the membrane hydraulic permeability in L/bar·m^2^·h, ∆P is the process’ transmembrane pressure (TMP) in bar, μ is the dynamic viscosity of the feed solution in bar·h, and $R_{m}$ is the intrinsic resistance of the membrane in m^2^. Alternatively, a follow-up expression can be used, where $V_{p}$ is the permeate volume obtained in L, $A_{m}$ is the membrane’s active area for filtration in m^2^, and t is time in h.

To assess the extraction efficiency of compounds in these processes, the rejection index $\left(R_{i}\right)$ (Equation (2)) can be used, which determines the membrane’s ability to prevent specific components from ending up in the permeate.

(2) $$R_{i}=\frac{C_{i,\;A}-C_{i,P}}{C_{i,A}}=1-\frac{C_{i,P}}{C_{i,A}}$$

where $C_{i,\;A}$ represents the concentration of species i in the feed solution and $C_{i,P}$ is the concentration of species i in the permeate. In this study, the rejection index refers to total polyphenol cumulative retention rates, based on tyrosol as reference the phenolic compound.

### 1.4. Membrane Fouling Models in UF

The particular challenge in using membranes, especially UF membranes, is their susceptibility to fouling effects and potential degradation if they are not adequately cared for and treated [29]. As a result, it is interesting to study the type of fouling that UF membranes exhibit when filtering any effluent [20,30,31]. Other authors have studied the fouling effects of polyphenols’ rich effluents [32,33,34].

**Figure 1:**
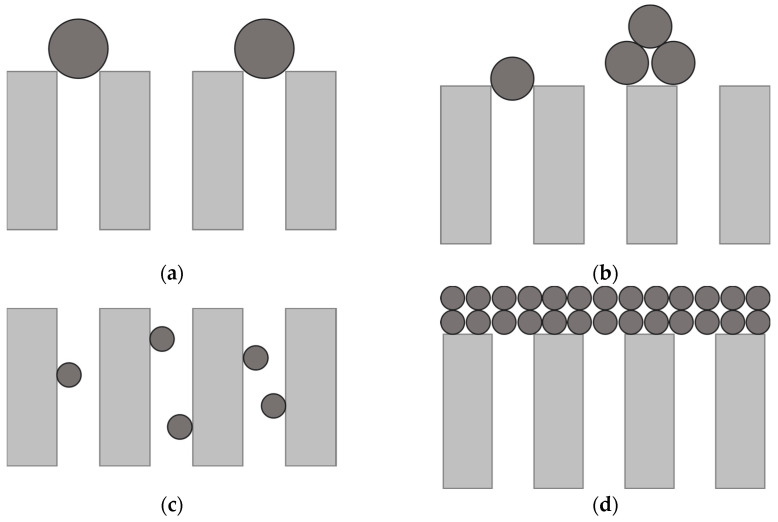
Categories of fouling developed by Hermia for ultrafiltration (UF) processes: (**a**) complete blocking, (**b**) intermediate blocking, (**c**) standard blocking, (**d**) cake layer formation.

Hermia [35] formulated four distinct empirical models, each elucidating a specific category of fouling: complete blocking, intermediate blocking, standard blocking and cake layer formation (Figure 1).

#### 1.4.1. Complete Blocking Model

The complete model applied to crossflow filtration assumes that molecules reaching the membrane surface and failing to pass through the pores completely block the membrane. It also posits that a molecule cannot overlap with a previously deposited particle. Given that the particle size is larger than the pore size in this type of blocking, it is blocked only on its surface, not within the membrane. The expression justifying this behavior is described in Equation (3).

(3) $$J_{v}=J_{Pss}+\left(J_{0}-J_{Pss}\right)\cdot e^{K_{c}\cdot J_{0}\cdot t}$$

where $J_{v}$ is the permeate flux in m/s, $J_{Pss}$ is the steady-state permeate flux in m/s, $J_{0}$ is the initial permeate flux in m/s, $K_{c}$ is the constant corresponding to the complete blocking model for crossflow filtration in 1/m, and t is time in seconds.

#### 1.4.2. Intermediate Blocking Model

As with the previously mentioned model, the intermediate blocking model also considers the possibility of particles depositing on top of each other. However, this fouling scenario occurs in cases where the particle size is similar to that of the pore, meaning that they can potentially obstruct it without entirely blocking it. This model is expressed as indicated in Equation (4).

(4) $$J_{v}=\frac{J_{0}\cdot J_{Pss}\cdot e^{K_{i}\cdot J_{Pss}\cdot t}}{J_{Pss}+J_{0}\cdot \left(e^{K_{i}\cdot J_{Pss}\cdot t}-1\right)}$$

where $J_{v}$ is the permeate flux in m/s, $J_{Pss}$ is the steady-state permeate flux in m/s, $J_{0}$ is the initial permeate flux in m/s, $K_{i}$ is the constant corresponding to the intermediate blocking model for crossflow filtration in 1/m, and t is time in seconds.

#### 1.4.3. Standard Blocking Model

The standard blocking model assumes that particles enter the membrane pores and become attached to the walls due to irregularities on the surface. Consequently, the volume of pores in the membrane gradually diminishes as filtration takes place. For this type of fouling to occur, the particles must be smaller than the pore size, meaning that fouling occurs within the pore. Furthermore, due to the previously mentioned fact, fouling becomes independent of the tangential flow velocity and the steady-state permeate term becomes null in extended experiments. The equation governing this model is as follows:

(5) $$J_{v}=\frac{1}{{\left(\frac{1}{J_{0}^{1/2}}+K_{s}\cdot t\right)}^{2}}$$

where $J_{v}$ is the permeate flux in m/s, $J_{0}$ is the initial permeate flux in m/s, $K_{s}$ is the constant corresponding to the standard blocking model for crossflow filtration in 1/s, and t is time in seconds.

#### 1.4.4. Cake Layer Formation

The last model capable of explaining a type of fouling in the membrane is based on the formation of a cake or gel layer on the membrane pores. In this case, particles do not enter the pores but instead form a layer on the membrane surface. The resulting expression to describe this phenomenon corresponds to Equation (6)

(6) $$t=\frac{1}{K_{gl}\cdot {J_{Pss}}^{2}\;}\cdot \ln\left[\left(\frac{J_{v}}{J_{0}}\cdot \frac{J_{0}-J_{Pss}}{J_{v}-J_{Pss}}\right)-J_{Pss}\cdot \left(\frac{1}{J_{v}}-\frac{1}{J_{0}}\right)\right]$$

where $J_{v}$ is the permeate flux in m/s, $J_{Pss}$ is the steady-state permeate flux in m/s, $J_{0}$ is the initial permeate flux in m/s, $K_{gl}$ is the constant corresponding to the cake layer formation blocking model for crossflow filtration in s/m^2^, and t is time in seconds.

### 1.5. Mathematical Model for NF Processes

NF has gained popularity in separation processes and is implemented in various industrial applications. The obtainment of a mathematical model which explains the behavior of such processes is essential to optimize them and reduce operational costs. In this study, Spiegler–Kedem simplified equations are used. They relate the transport of solvent and solute in order to calculate the permeate flux of an NF membrane filtration process ($J_{v}$, in L/m^2^·h). In addition, the concentration polarization and the elimination of the repulsion due to an electric charge on the membrane surface are considered. The resulting equation, developed by other authors [36,37], is as follows:

(7) $$J_{v}=L_{p}\cdot \left[∆P-\sigma \cdot R\cdot T\cdot \left(C_{f}-C_{p}\right)\cdot \exp\left(\frac{J_{v}}{k}\right)\right]$$

where $L_{p}$ is the hydraulic permeability in L/bar·m^2^·h, ∆P is the TMP in bar, and σ is the reflection coefficient, which depends on the membrane’s structure, composition, and affinity with the solution it is in contact with. R is the universal gas constant in J/mol·K, T is the process temperature in K, $C_{f}$ and $C_{p}$ are the polyphenols’ concentrations in the feed solute and permeate solutions in mol/L, and k is the mass transfer coefficient parameter in L/m^2^·h.

Overall, this study focuses on the implementation of membrane technology for the concentration of polyphenols, along with an investigation of the associated cleaning procedure, an overlooked yet critical aspect for industrial application. It is anticipated that the findings of this research will significantly contribute to the implementation of this technology by offering practical and efficient conditions for polyphenol concentration and membrane efficacy maintenance in industrial applications.

## 2. Materials and Methods

### 2.1. Determination of Phenolic Compounds

The method employed for the quantification of polyphenols involves a colorimetric technique using the Folin–Ciocalteu reagent (FCR), a mixture of phosphomolybdic and phosphotungstic acids that, upon reduction, takes on a bluish tone that can be measured through the absorbance of the sample at a wavelength of 765 nm [38]. Due to the large amount of existing phenolic compounds, the concentration of tyrosol in mg/L is used as reference [27].

Regarding the material used to analyze total phenolic compounds in the samples, the equipment employed for absorbance reading was a Thermo Spectronic Helios δ spectrophotometer manufactured by Thermo Fisher Scientific (Waltham, MA, USA).

### 2.2. Wine Lees Residues

The wine lees residues used as the feed for filtration were obtained from a winery located in the Valencian Community, Spain, generated in 2022. The wine lees exhibited a uniform garnet color. Additionally, the presence of solid particles corresponding to the sediment in the lees was noteworthy. Consequently, a pretreatment stage was necessary to remove larger colloids and thus prevent excessive fouling of the membranes.

The wine lees utilized were characterized by analysing the pH, conductivity, chemical oxygen demand (COD), total suspended solids (TSS), volatile suspended solids (VSS), fixed suspended solids (FSS), and total polyphenol content. These parameters are shown in Table 1.

### 2.3. Membranes and Experimental Set-Up

In this study, an INSIDE CéRAM™ membrane manufactured by TAMI Industries (Nyon, France) was used for the UF pretreatment step, and a FilmTech™ NF270-2540 polymeric membrane manufactured by DuPont de Nemours, Inc. (Wilmington, DE, USA) for the NF process. The specifications of each can be found in Table 2.

**Figure 2:**
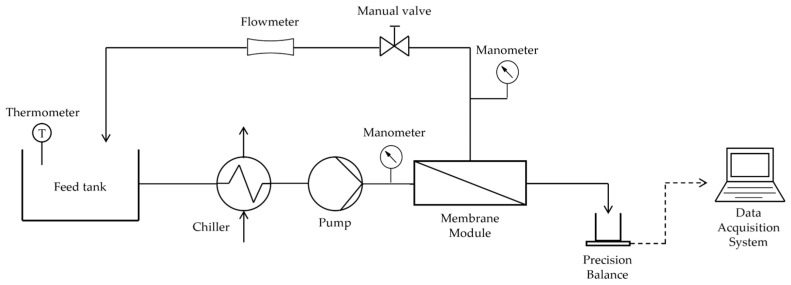
Experimental setup for UF and NF pilot plants.

The two differentiated pilot plants used for polyphenol extraction in UF and NF processes followed the same system illustrated in Figure 2.

This system includes a Kern PLS balance of 4200 g capacity with a resolution of 0.01 g, a PolyScience (Niles, IL, USA) refrigeration unit, and a Testo 922 thermocouple with a resolution of 0.1 °C. Likuid Nanotek S.L. (San Sebastián, Spain) assembled the UF plant with a 1178 mm long tubular ceramic membrane module, whereas the crossflow membrane module utilized in the NF plant was a CF042SS CELL model manufactured by Sterlitech (Auburn, WA, USA).

The permeate resulting from the filtration sessions was collected in a container, and the balance continuously recorded its weight variation. These data were subsequently collected on a laptop via LabView. The processing of the gathered data was then carried out using Matlab R2021b.

### 2.4. Pretreatment and Conservation of Wine Lees

The wine lees studied were kept at room temperature before the filtration experiments. A filtration process was applied before the experiments to remove larger particles in the treated wine lees waste. Paper filters of 100 and 5 μm were initially employed, while 20 μm thread filters were used in cases where a mold layer had formed on the liquid surface.

Furthermore, due to temperature sensitivity, the permeate solutions extracted were stored in a refrigerator at approximately 3 °C. This approach aimed to reduce the formation of a mold layer on the surface of the mixture.

### 2.5. UF and NF Membrane Permeability Characterisation Experiments

The experiments to determine the permeability of each membrane were conducted using distilled water. Initially, the distilled water was circulated throughout the circuit with the cooling equipment operational until a stable and constant temperature for the test was achieved. The NF membrane used was previously compacted at 14.5 bar for 30 min.

The UF membrane permeability experiments were conducted by modifying the temperature operating conditions, alternating between 15, 20, 25, and 27 °C while maintaining the TMP at 2 bar and a constant feed flow of 50 L/min. The NF membrane permeability experiments were conducted at a temperature of 20 °C and a TMP of 4.5, 7.0, 9.5, 12.0, and 14.5 bar. The resulting concentrated flow rate was maintained at 5.75 L/min, measured using the flowmeter present in the plant.

### 2.6. UF and NF Filtration Experiments with Wine Lees

Regarding the experimentally conducted filtration tests with wine lees on the UF plant, all tests maintained a TMP of 2 bar at a constant temperature of 20 °C. The NF filtration experiments were conducted at a TMP of 9.5 bar, a continuous feed temperature of 20 °C, and a rejection flow rate of 5.75 L/min.

The wine lees feed solution was constantly recirculated in the UF pretreatment step to maintain the same feed properties. The solution was poured into the tank and allowed to reach the specified temperature. A total of six experiments were recorded. The initial test was run to observe the permeate flux reduction throughout the experiment. Posterior tests included two filtration tests after the initial run to observe the membrane’s behavior without intermediate cleaning. Afterwards, two filtration tests were performed after cleaning with distilled water to verify the adequacy of water cleaning to recover the permeate flux. Finally, a filtration test was conducted after chemical washing with caustic soda to study the recovery of permeate flux upon the filtration of wine lees.

The permeate that resulted from the UF step was used as the feed solution for the NF step. This solution was also recirculated to conserve the filtering conditions. Once the desired conditions were attained, the test was initiated. Six experiments were conducted in the NF plant. The first test was run to perceive the permeate flux evolution when filtering the permeate solution previously mentioned. A posterior experiment was performed without intermediate cleaning to observe the behavior of the resulting permeate flux. Afterwards, two sets of two identical filtration experiments were run. The first was a filtration test in which prior chemical washing was performed due to the significant permeate flux loss. The second part was a filtration experiment in which no intermediate cleaning was performed to analyze the change in permeate flux. The calculation of permeate flux followed the procedure described in Section 2.3.

Throughout the tests, samples of the feed and permeate were taken to verify their polyphenol concentration using the Folin–Ciocalteu method.

### 2.7. Cleaning Experiments

Two different cleaning procedures were performed to clean both UF and NF membranes. Firstly, distilled water was initially used in recirculation to remove surface fouling from the membrane for 2–3 h. In cases where fouling was particularly pronounced, 1% NaOH solutions were prepared and circulated through the filtration system for 2–3 h intervals.

Following the 2–3 h recirculation cleaning with caustic soda, the system was left at rest within the circuit for 24 h. Subsequently, a rinse with distilled water was performed until a neutral pH in both the UF and NF systems was reached.

## 3. Results and Discussion

### 3.1. UF and NF Membrane Characterisation

The mean permeate flux values obtained in each experiment at different temperatures regarding the UF ceramic membrane used can be observed in Table 3. They show that the permeate flux increases with temperature, which is a logical behavior in membrane filtration processes.

**Figure 3:**
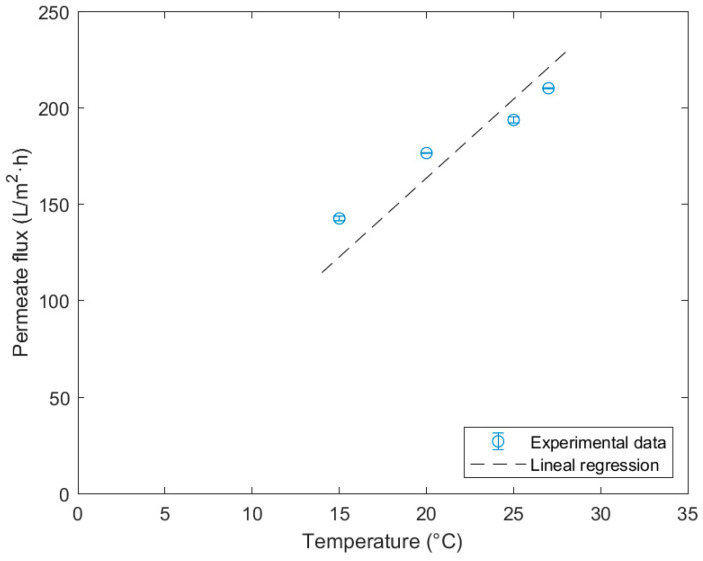
Determination of the hydraulic permeability of 15 kDa ceramic membrane at 2 bar.

Afterwards, each value is taken, and a lineal regression is applied, plotting the permeate flux versus temperature (Figure 3). This adjustment is made by forcing the intersection through the origin, thus determining the water permeability of the membrane at a fixed TMP of 2 bar with a value of 8.18 ± 0.37 L/°C·m^2^·h and a R^2^ of 0.994.

As is evident, the resulting permeability parameter exhibits a regression coefficient of 0.994, indicating a good fit. Regarding the resulting value, information has yet to be found in the literature employing this type of membrane in similar experiments, making it impossible to validate the obtained result. However, studies that used ceramic membranes of 15 kDa, similar to the one used in this work [39,40,41], show flux values inferior to the ones obtained experimentally in this study, presenting values ranging from 30 L/m^2^·h (at 1 bar of TMP and 20 °C) to 40 L/m^2^·h and 84 L/m^2^·h (at 2 bar and 20 °C and 25 °C, respectively). The permeability experiments in NF resulted in the permeate values compiled in Table 4.

**Figure 4:**
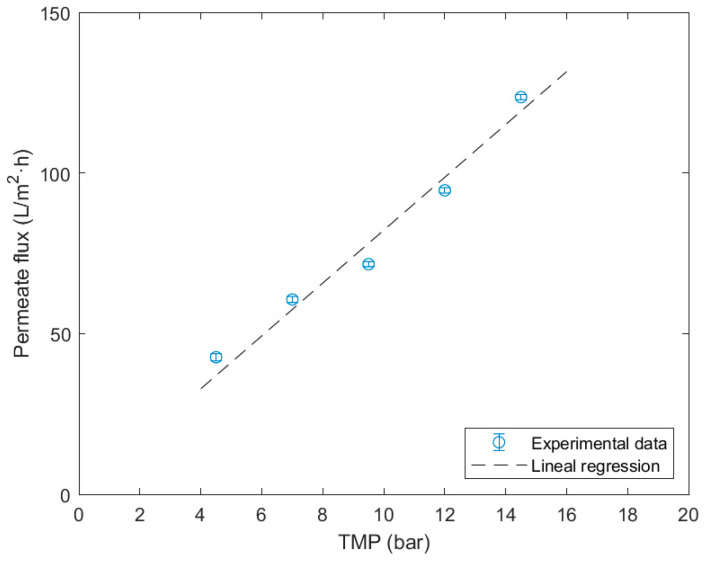
Determination of the hydraulic permeability of NF270 membrane at 20 °C.

A linear regression is performed, plotting the permeate flux versus the TMP (Figure 4). Once again, this adjustment is made by forcing the intersection through the origin, determining the water permeability of the membrane at a fixed temperature of 20 °C, with a value of 8.23 ± 0.24 L/bar·m^2^·h and a R^2^ of 0.997.

The resulting permeability parameter presents an elevated regression coefficient of 0.997, which indicates a good fit. This value is similar to those reported by other authors [27,42,43,44], maintaining the same order of magnitude. Values of permeability 8.15 L/bar·m^2^·h were observed and the same operating conditions, whereas other experiments presented values of 7.04 and 8.7 L/bar·m^2^·h at an operating temperature of 25 °C. These differences could be explained by the volumetric flow applied in the experiment, as they were inferior to the experiments conducted in this study.

### 3.2. Polyphenols Selectivity in Sequential Membrane Filtration Process

Throughout the filtration experiments, samples were taken from the resulting permeate solution obtained in each step and analyzed using the Folin–Ciocalteu method. The total polyphenols rejection results of each membrane are shown in Table 5.

As observed, the UF pretreatment step, filtering the same wine lees solution, constantly exhibits similar behavior across the experiments, yielding an average rejection of 54.2 ± 3.0%. This indicates that approximately half of the polyphenols in the wine lees have a molecular size larger than 15 kDa. Wine lees and wine present large particles, such as proteins and polysaccharides, in solution, generally larger than 20 and 100 kDa, respectively [45]. This implies that the UF step eliminates these compounds from the permeate solution rich in polyphenols, as their molecular weight is inferior to approximately 3 kDa [46]. However, it is noteworthy that some polyphenols could be trapped in such particles, therefore giving such polyphenol rejection values.

The rejection values obtained in the NF are very similar. On average, a rejection of 90.1 ± 1.1% is determined. This implies that a significant proportion of the polyphenols in the NF feed cannot pass through the 340 Da polymeric membrane and are reintroduced into the feed. In this case, the membrane is more selective to certain polyphenols in the filtered solution. Phenolic compounds, such as procyanidins or malvidin, are detected in the most significant quantities in wine, up to 500 and 4000 mg/L [47]. Their molecular weight ranges between 463.41 and 866.77 Da [46], which the membrane would reject. As a result, the number of polyphenols that are filtered and end up in the permeate is reduced substantially.

### 3.3. UF and NF Membrane Recovery Analysis

To study the effectiveness and state of the membranes used in the UF and NF processes, several experiments were conducted using the same feed solution in each step to analyze the fouling effect in each membrane as well as how the permeate flux can be recovered after the filtration of said solutions.

**Figure 5:**
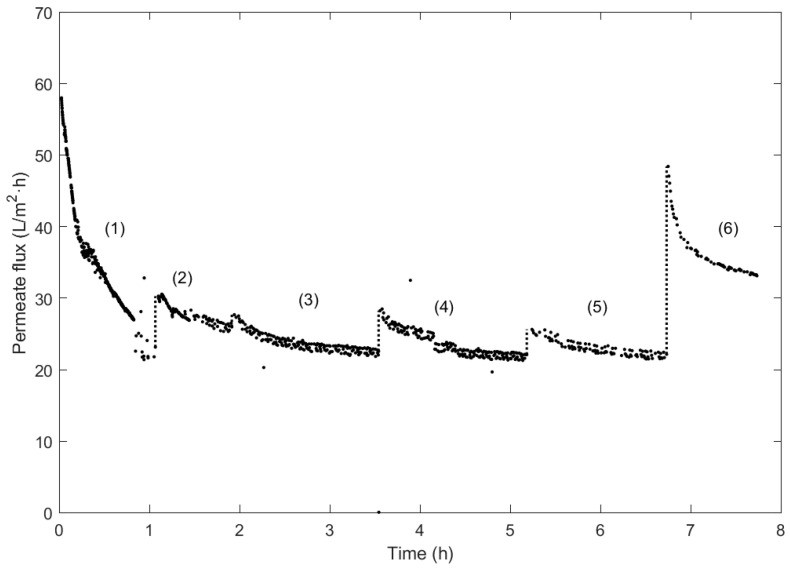
Permeate flux measurements of UF cycles with the recirculation of wine lees at 20 °C and a TMP of 2 bar.

Regarding the UF filtration experiments, the resulting permeate flux in each is represented in Figure 5.

Test (1) corresponds to the first filtration experiment of the lees, where it can be observed that the flux decreases drastically from 60 L/m^2^·h to approximately 20 m^2^·h. The filtration process was paused between filtration tests (2) and (3). Considering the initial flux value obtained in the experiment (1), the recovery of the membrane was calculated. In this case, it is observed that the membrane recovers up to 52.31% in test (2)

Afterwards, a cleaning with distilled water was performed to observe the membrane recovery after two filtration trials without intermediate cleaning. The subsequent results of the filtration correspond to test (4). In this case, the recovery increases to 48.79%, indicating that the water cleaning can remove some of the residual particles present in the membrane after two filtration processes. Despite recovering some of the initial permeate flux, more would be needed to consider this cleaning step effective in recovering the membrane.

Regarding test (5), a distilled water wash was performed before this trial to verify whether the permeate flux could decrease below the initially observed 20 m^2^·h after the solution filtration. In this case, the flux reached a similar flux value, and the obtained recovery is 44.28%. It is observed that the second water cleaning was not as beneficial as the first water cleaning due to the decrease in the membrane recovery value from 52.31% to 44.28%.

Due to the decrease in flux obtained in the trial compared to the initial one in test (1), a chemical wash with 1% NaOH was subsequently performed, as previously explained, to verify if it was possible to recover the initial flux fully.

With the collected data from test (6), the effectiveness of the chemical cleaning of the membrane is evident, as a recovery of 83.68% has been achieved. Consequently, periodic cleaning with caustic soda allows the wine lees filtration process to be repeated. The flux difference between the first and last tests recorded could be explained by the large molecules in the solution attached to the membrane breaking down into smaller ones after the washings and filtration cycles.

It is worth noting that the difference between the initial flux recorded and that obtained after chemical cleaning indicates that a percentage cannot be recovered due to the generation of irreversible fouling. This would require a more thorough washing process, increasing the maintenance cost. The flux recovery values obtained for each test are compiled in Table 6.

**Figure 6:**
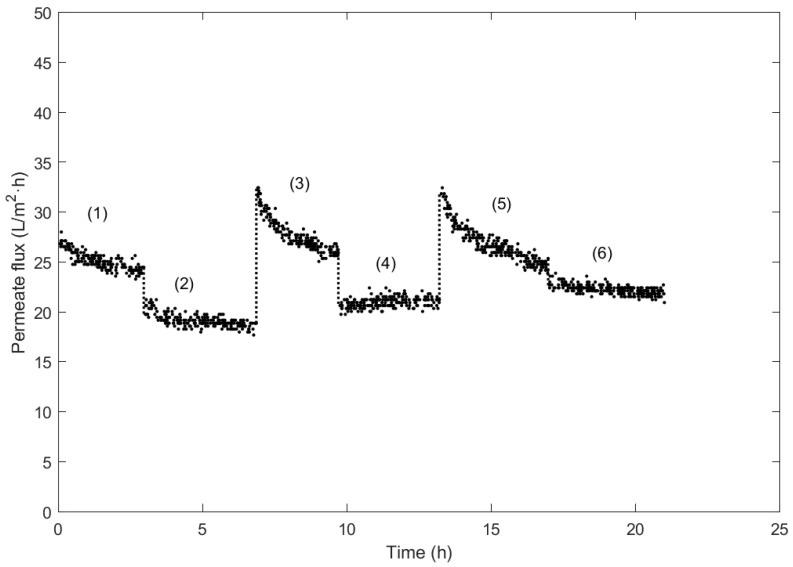
Permeate flux measurements of NF cycles with the recirculation of the UF permeate solution at 20 °C and a TMP of 9.5 bar.

The NF filtration experiments are shown in Figure 6, in which the filtration of the UF permeate is constantly recirculated through the system.

Test (1) corresponds to the first filtration process of the permeate extracted from the previous UF stage. A decrease in the resulting permeate flux is observed, from 27 L/m^2^·h to approximately 25 L/m^2^·h after 2 h of filtration.

Once the initial test was completed, the subsequent filtration was carried out, simulating a discontinuous filtration process without a prior washing and after turning off the pump to check whether the permeate x would continue to decrease from the flux value measured in test (1). As seen in test (2), a significant decrease in permeate flux occurs, reaching 19 L/m^2^·h.

Following the initial tests, cleaning with caustic soda was necessary upon the reduction from 27 to 19 L/m^2^·h. In this way, the efficacy of caustic soda can be verified. The effect can be visualized in test (3), where the flux is considerably increased to 33 L/m^2^·h, surpassing the flux measured in test (1).

With the completion of test (3), feed filtration was again carried out without prior cleaning. As a result, the data corresponding to test (4) are obtained. They show a dissimilar behavior compared to the registered tests, where the flux initially increases slightly. This event can be justified due to the effect of temperature, where, initially, the feed was at a lower temperature. As the test progressed, the temperature stabilized at the desired value. With this test, the importance of temperature in membrane filtration processes can be appreciated.

Before test (5), the membrane was cleaned with caustic soda. As a result, a new increase of similar proportions to that recorded in test (3) is evident. This phenomenon is caused by the caustic soda on the membrane, which could break down part of its dense layer, and in the membrane module, meaning a higher permeate flux can be extracted. Nevertheless, this is only observed in the first cleaning cycle, as posterior chemical cleaning cycles did not further improve the permeate flux obtained.

Finally, in test (6), no posterior cleaning step was performed, resulting in a reasonably stabilized flux of 22 L/m^2^·h, similar to the one recorded in test (4). Therefore, chemical cleaning provokes the permeate flux to reach stable values of 20–22 L/m^2^·h. This behavior contrasts with that observed in previous runs due to the plant being idle for a shorter period between tests.

Overall, it can be observed that chemical cleaning is required to maintain a high permeate extraction rate, which implies that a previous filtration step would be necessary to remove as many large molecules as possible to ensure the lifespan of the membrane can be prolonged. The resulting values of membrane recovery after both cleanings compared to the initial flux of test (1) are collected in Table 7.

To study the effectiveness of each washing process, the permeate flux using distilled water was reobtained after each cleaning step. Using the initial flux of the membrane before the filtration experiments, the recovery of the membrane is calculated, resulting in the values presented in Table 8 for both UF and NF processes.

As can be seen, cleaning with NaOH offers the best results in terms of membrane recovery in both filtration steps, recovering up to 95% and 135% of the original permeate flux in the UF and NF processes, respectively. However, cleaning with NaOH results in permeate flux in the NF membrane being significantly higher than the measured initial flux due to a possible damaging effect on the membrane. In contrast, cleaning with distilled water is less effective, as only 36% of the original permeate flux can be recovered with this type of wash in the UF step and up to 75% in the NF step.

### 3.4. Fouling Identification Type in UF Membrane with Wine Lees Filtration

**Figure 7:**
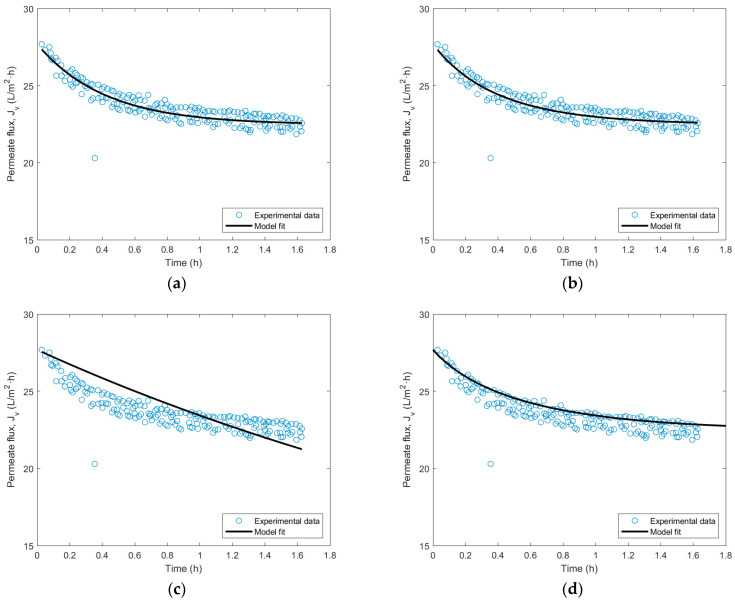
Mathematical adjustments of Hermia’s fouling crossflow blocking parameters to experimental wine lees filtration data. (**a**) complete blocking model adjustment. Fit = 85.09%, (**b**) intermediate blocking model adjustment. Fit = 96.72%, (**c**) standard blocking model adjustment. Fit = 96.77%, (**d**) cake layer formation model adjustment. Fit = 53.82%.

Membrane fouling in UF is one of the main problems with such processes, as it reduces the efficiency of the membrane after extended filtration periods. Therefore, it is interesting to study the current crossflow fouling models to observe the behavior of the membrane upon the filtration of wine lees. After conducting fouling tests with a 15 kDa ceramic membrane, the resulting fits are showcased for each of the four models proposed in Figure 7.

Out of the four fouling models, it is noticeable that the complete and intermediate blocking models are the ones that best fit the experimental data, with a fitting parameter of 85.09 and 96.72%, respectively. On the contrary, the standard blocking model does not predict the experimental behavior adequately, although it presents a fitting parameter of 96.77%. Finally, the cake formation blocking model seems to fit satisfactorily at the beginning of the test. Still, the dispersion of subsequent data points causes the fit to be less adequate than the first two, having a value of 53.82%. The fitting parameters obtained for each model are compiled in Table 9.

In conclusion, it can be argued that the fouling type experienced by a UF 15 kDa ceramic membrane subjected to a wine lees filtration process may involve complete or intermediate pore blocking. This interpretation agrees with the fouling type identified by other authors when filtering clarified grape juice using a tubular ceramic UF membrane [48] or when treating winery wastewaters with a polymeric membrane [49].

### 3.5. Fitting of the Spiegler–Kedem NF Model

**Figure 8:**
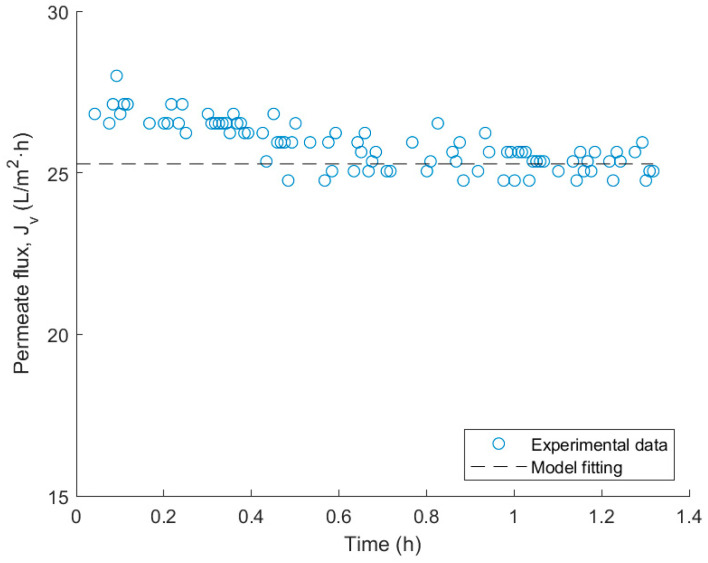
Experimental and theoretical permeate flux data obtained after fitting the Spiegler-Kedem model.

The mathematical model corresponding to expression (7) is fitted in this section. Since the permeability has been previously determined, this value will be used in the fitting process. Additionally, to obtain the mass transfer coefficient (k), a value T of 293.15 K and ΔP of 9.5 bar are considered. The parameter σ is attributed a value of 0.999, considering that the membrane is almost perfectly selective to polyphenols. Concentrations of feed ($C_{f}$) and permeate ($C_{p}$) are set at 950 and 80 mg of Tyrosol eq/L, respectively, as obtained during experimental tests. The permeate flux ($J_{v}$) is assigned a value of 25.28 L/m^2^·h, as experimentally it was observed that it reached this steady value, thus, this would adequately represent the filtration process. The adjusted mass transfer coefficient is 6.74 L/m^2^·h. In Figure 8, the steady-state permeate flux obtained with the model fitting is represented alongside the flux obtained in the filtration test.

Other authors have identified the mass transfer coefficient of polyphenols in winery wastewaters filtration. Using the same NF membrane, values of 135 L/m^2^·h have been identified with the filtration of wastewater originating from the second racking from red wine production [44], and 6.2 L/m^2^·h with the direct filtration of wine lees [27]. However, the difference in nature of the analyzed residues complicates a comparison with other studies.

### 3.6. Polyphenol Content in Wine Lees Analysis for Industrial Application

Working with polyphenols industrially requires control over their content in solution, which can be altered due to factors such as polyphenol degradation. Other authors have previously studied and identified such behavior [50,51], in which temperature causes molecules to degrade after prolonged periods. In this study, polyphenol concentration in the wine lees solution was measured throughout the filtration process, before and during the filtration experiments, in which temperature was kept constant, as indicated previously. The results are identified in Table 10.

It can be observed that before the filtration experiments, the concentration was maintained steadily. This implies that the polyphenols present in wine lees solution do not degrade over time after being kept in the storage conditions established previously. In addition to this, the experiments were done over a period of one month. However, once the filtration process was initiated, there was an evident reduction in the concentration of phenolic compounds in the feed solution. This could be explained due to the fouling effect on the membrane. Large particles, such as proteins, would have attached to the membrane alongside small polyphenol molecules. They could be trapped inside these large macromolecules, and the polyphenol concentration could decrease. Despite this, the concentration of the feed solution after its filtration was maintained steadily as well, meaning that this reduction would only happen after the first filtration of the wine lees and once membrane fouling has occurred.

## 4. Conclusions

The effectiveness of membrane separation technology for extracting phenolic compounds from wine lees waste from the wine production industry has been studied. It proves an adequate process to reduce the waste generated by the wine industry and revalorize residues by extracting high added-value components.

The ceramic and polymeric membranes used for polyphenol extraction have been experimentally characterized. The first one, according to the process temperature at a constant 2 bar TMP, with a permeability of 8.18 ± 0.37 L/°C·m^2^·h. The polymeric membrane used in NF presented a permeability of 8.23 ± 0.24 L/bar·m^2^·h at a constant 20 °C. The impact of filtering such waste has also been examined, revealing that chemical cleaning results in better membrane recovery and increased reusability, reaching a 92.5 ± 0.3% recovery average in UF and a 134.8 ± 0.7% recovery average in NF.

Regarding the phenolic content after filtration processes, it has been concluded that passage through a 15 kDa pore-sized membrane retains an average of 54.2 ± 3.0% of phenolic compounds in vinasse waste. Using a membrane allowing the passage of particles smaller than 340 Da to treat the permeate from the previous filtration, it has been determined that 90.1 ± 1.1% of phenolic compounds are too voluminous, and only a tiny portion of these compounds can be extracted. A UF pretreatment stage of wine lees before NF has been found to benefit the overall process. This results in a significant reduction in membrane fouling in the latter NF stage and an increase in the extraction efficiency of desired compounds.

Furthermore, fouling models developed by Hermia have been studied in the 15 kDa ceramic membrane used in the UF step. The analysis resulted in an intermediate or complete pore blocking of the membranes, as they showed a better fit. The complete blocking model presented a $K_{c}$ constant of 86.5 ± 5.1 1/m with an 85.09% fit. The intermediate blocking model showed a better fitting parameter of 96.72% and a $K_{i}$ parameter of 95.3 ± 6.2 1/m. Standard blocking and cake layer formation models did not appropriately fit the experimental data, despite acquiring a fitting parameter of 96.77% and 53.82%, respectively. $K_{s}$ parameter resulted in 87.3 × 10^−4^ ± 3.8 × 10^−4^ 1/m and $K_{gl}$ a value of 1.07 × 10^7^ ± 0.04 × 10^7^ s/m^2^. This agrees with the filtration and cleaning study of the UF process, as a thorough membrane cleaning was necessary to regain the initial permeability due to the fouling effect.

The k parameter from Spiegler–Kedem was obtained from experimental data by solving the suggested equation. The mass transfer parameter resulted in a value of 6.74 L/m^2^·h. However, further studies need to be made to verify the adequacy of this model to a wine lees NF process.

Finally, the polyphenol content in wine lees was measured. The polyphenol concentration remains stable after storing the feed solution at room temperature. The possible reduction in its content could be due to small phenolic compounds being stuck in macromolecules, which are rejected by the ceramic membrane and get stuck on its surface, further increasing the fouling effect on the membrane.

## Figures and Tables

**Table 1:** Characterisation of wine lees.

Parameter	Value
pH	3.75 ± 0.01 ^1^
Conductivity (mS/cm)	4.8 ± 0.9
COD (g/L)	176.2 ± 0.5
Turbidity (NTU)	25,275 ± 301
TSS (g/L)	66.2 ± 4.7
VSS (g/L)	56.6 ± 5.2
FSS (g/L)	17.2 ± 2.8
Total polyphenols (mg Tyrosol eq/L)	2320 ± 109

^1^ Standard deviation.

**Table 2:** Specifications of commercial membranes of UF and nanofiltration (NF).

Membrane	INSIDE CéRAM™	NF270
Membrane type	Ultrafiltration	Nanofiltration
Material	TiO_2_	Thin film composite polyamide
MWCO (Da)	15,000	340
Active area (cm^2^)	2500	42
Stabilized salt rejection (%)	- ^a^	>97.0
Free chlorine tolerance (ppm)	-	<0.1
Maximum operation pressure (bar)	4	41
Maximum operating temperature (°C)	350	45
pH range	2–12	3–10 (Continuous operation) 1–12 (Short-term cleaning)

^a^ Not available.

**Table 3:** Permeate flux values for experiments with distilled water in UF with 15 kDa ceramic membrane at a TMP of 2 bar.

Temperature (°C)	$J_{v}$ (L/m^2^·h)
15	142.7 ± 1.2
20	176.7 ± 0.2
25	193.8 ± 1.5
27	210.2 ± 0.2

**Table 4:** Permeate flux values for experiments with distilled water in NF with NF270 at a constant temperature of 20 °C.

TMP (bar)	$J_{v}$ (L/m^2^·h)
4.5	42.7 ± 1.1
7.0	60.7 ± 1.0
9.5	71.7 ± 0.8
12.0	94.7 ± 0.8
14.5	123.9 ± 0.8

**Table 5:** Total polyphenols rejection values of UF and NF experiments conducted in the wine lees sequential filtration process.

Filtration Step	Experiment	Rejection (%)
UF	1	57.6 ± 6.5
	2	52.1 ± 4.8
	3	53.0 ± 4.2
NF	1	90.0 ± 1.2
	2	91.2 ± 0.6
	3	90.4 ± 1.0
	4	90.4 ± 1.2
	5	88.1 ± 2.0
	6	90.8 ± 0.8

**Table 6:** Flux recovery values for each filtration experiment with wine lees in the UF process.

Experiment	Recovery (%)
2	52.31 ± 0.03
4	48.79 ± 0.03
5	44.28 ± 0.02
6	83.68 ± 0.03

**Table 7:** Flux recovery values for each filtration experiment with wine lees in the NF process.

Experiment	Recovery (%)
3	120.91 ± 0.08
5	118.71 ± 0.08

**Table 8:** Permeate flux and recovery values with distilled water initially and after each UF and NF process cleaning step.

Filtration Step	Experiment	$J_{v}$ (L/m^2^·h)	Recovery (%)
UF	Initial flux	170.6 ± 0.3	-
	Cleaning with NaOH	157.8 ± 0.2	92.5 ± 0.3
	Cleaning with distilled water	61.4 ± 0.2	36.0 ± 0.2
NF	Initial flux	71.8 ± 0.9	-
	Cleaning with NaOH	96.7 ± 1.0	134.8 ± 0.7
	Cleaning with distilled water	53.8 ± 2.4	75.0 ± 0.3

**Table 9:** Fitting parameters of Hermia’s fouling models in UF process with wine lees.

Fouling Model	Fitting Parameter	Value
Complete blocking	$K_{c}$ (1/m)	86.5 ± 5.1
Intermediate blocking	$K_{i}$ (1/m)	95.3 ± 6.2
Standard blocking	$K_{s}$ (1/s)·10^4^	87.3 ± 3.8
Cake layer formation	$K_{gl}$ (s/m^2^)·10^−7^	1.07 ± 0.04

**Table 10:** Total polyphenols variation in wine lees.

Sampling Time	Experiment	Total Polyphenols (mg Tyrosol eq/L)
Before filtration	1	2320 ± 109
	2	2482 ± 304
	3	2231 ± 246
After filtration	1	1585 ± 95
	2	1588 ± 81

## Data Availability

The data presented in this study is available upon request from the corresponding author.

## Abbreviations

COD	Chemical Oxygen Demand
FCR	Folin-Ciocalteu Reagent
FSS	Fixed Suspended Solids
MF	Microfiltration
NF	Nanofiltration
RO	Reverse Osmosis
TMP	Transmembrane Pressure
TSS	Total Suspended Solids
UF	Ultrafiltration
VSS	Volatile Suspended Solids

## Nomenclature

$A_{m}$	Membrane’s active area, m^2^
$C_{A}$	Concentration of polyphenols in feed solution, mol/L
$C_{P}$	Concentration of polyphenols in permeate solution, mol/L
$J_{0}$	Initial permeate flux, m/s
$J_{Pss}$	Steady-state permeate flux, m/s
$J_{v}$	Permeate flux, m/s
k	Mass transfer coefficient parameter in L/m^2^·h.
$K_{c}$	Complete blocking model constant for crossflow filtration, 1/m
$K_{gl}$	Cake layer formation blocking model constant for crossflow filtration, s/m^2^
$K_{i}$	Intermediate blocking model constant for crossflow filtration, 1/m
$K_{s}$	Standard blocking model constant for crossflow filtration, 1/s
$L_{p}$	Membrane hydraulic permeability, L/bar·m^2^·h
R	Universal gas constant, J/mol·K
$R_{i}$	Rejection index
$R_{m}$	Intrinsic resistance of the membrane, m^2^
T	Temperature, K
t	Time, s
$V_{p}$	Permeate volume obtained, L
Greek
∆P	Transmembrane pressure, bar
μ	Dynamic viscosity, bar·h
σ	Reflection coefficient
